# Supplementary material for: The impact of errors in medical certification on the accuracy of the underlying cause of death
Source: PLoS One. 2021 Nov 8;16(11):e0259667. doi: 10.1371/journal.pone.0259667 (PMC8575485; doi:10.1371/journal.pone.0259667)
Supplement: S1 Text — (DOCX) [file pone.0259667.s001.docx]

**S1 Text. International Form of the Medical Certificate of Cause of Death (MCCOD)**

The cause of death section of the international form consists of two parts (S1 Fig). The first part (Part 1) is for reporting the sequence of events leading to death, proceeding backwards from the final disease or condition resulting in death. Each condition in Part 1 should cause the condition above it. A specific cause of death should be reported in the last entry (lowest used line) in Part 1 and there should be no ambiguity about the aetiology of this cause. This is usually the underlying cause of death selected for tabulation.(1) If there is only one cause, it is entered at line 1a. Where two or more conditions must be recorded, the certifying officer should record the sequence of events leading to death. Each event should be recorded on a separate line in reverse order.(2)

**S1 Fig. International Form of the Medical Certificate of Cause of Death (MCCOD)**


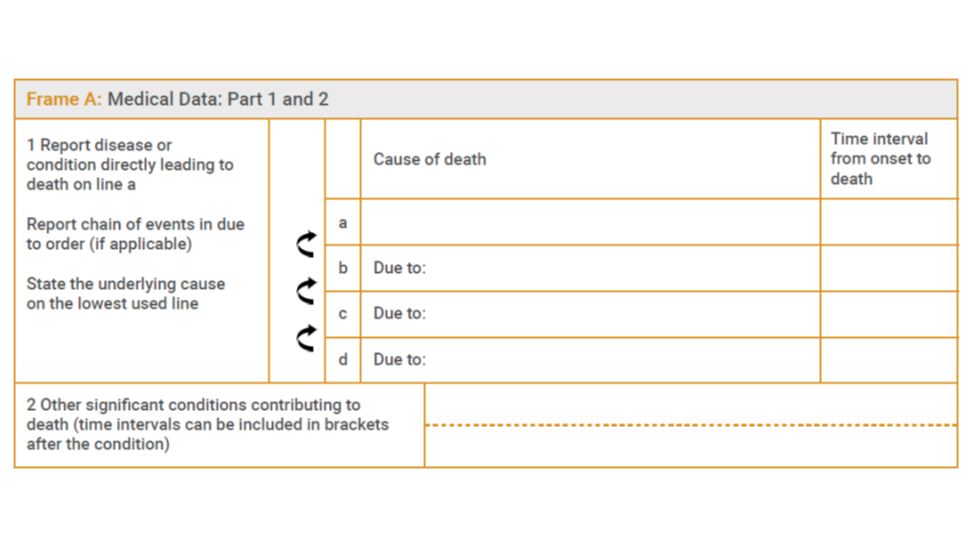


**References**

1. NCHS. Report of the second workshop on improving cause-of-death statistics. Virginia Beach, Virginia, April 21–23 Hyattsville, MD 1991. 2004.

2. WHO. International statistical classification of diseases and related health problems. - 10th revision. 05th edition ed2016.
