# Supplementary material for: The impact of errors in medical certification on the accuracy of the underlying cause of death
Source: PLoS One. 2021 Nov 8;16(11):e0259667. doi: 10.1371/journal.pone.0259667 (PMC8575485; doi:10.1371/journal.pone.0259667)
Supplement: S2 Text — (DOCX) [file pone.0259667.s002.docx]

**S2 Text. Certification errors**

1. **Reporting multiple causes in a single line of part 1**

The WHO’s death certification guidelines state that only one cause should be recorded per line in a death certificate.(1) When more than one cause is reported on a single line, it makes it difficult for coders to establish the sequence of events leading to death, thus selecting the correct underlying cause of death would be more difficult.

1. **Incorrect or clinically improbable sequencing of causes in Part 1 of the death certificate**

Mortality statistics are based on the underlying cause of death, which is the condition or injury that initiated the sequence of events that led directly to death. The guidelines state that the certifying doctor should identify a sequence of events leading to death, and document these in the death certificate. When a clinically improbable sequence of events is recorded, the selection of the correct underlying cause of death is more difficult.

1. **Reported underlying cause of death in the lowest used line of Part 1 is ill-defined**

Entering ill-defined or vague conditions on death certificates greatly diminishes their value for public health and does not provide any information for decision-makers to guide them in designing preventive health programs. Such conditions are usually coded to unusable (or ‘garbage’) codes, which belong to four main types:(1-3)

1. Symptoms and signs (e.g. fever, headache, backache, enlarged liver)

2. Intermediate causes (e.g. septicemia, pathological fracture, pneumonitis, secondary hypertension etc.)

3. Modes of dying (e.g. cardiac or respiratory arrest)

4. Unspecified causes within a larger death category (e.g. ill-defined site of cancer or injury, congenital heart disease, respiratory infection, cardiovascular disease etc.).

1. **Use of non-standard abbreviations**

Any abbreviation used in Part 1 or Part 2 is considered an error because the coders may misinterpret the non-standard abbreviation and code the cause to a non-relevant code.

1. **Illegible entries**

Death certificates need to be completed clearly so that coders and other users can read the information provided in the death certificate. Illegible handwriting makes it hard for coders to correctly identify the stated condition even if the death certificate contains no other errors.(1)

1. **Incorrect or absent time intervals**

The column on the right-hand side of Part 1 of the death certificate is for recording the approximate time interval between the onset of the condition and the time of death. The time interval should be entered for all conditions reported on the death certificate, in both Part 1 and Part 2 (4). Absent or incorrect time intervals can affect coding and the selection of the correct underlying cause of death.(1)

1. **Reporting competing causes in Part 1**

Reporting two or more mutually exclusive yet possible causes as the underlying cause of deaths in Part 1 of death certificate. This can lead to selection of an incorrect underlying cause of death by the coders. For the purposes of this study, if two competing causes were reported on a single line of Part 1, then the error was regarded as “Reporting multiple causes in a single line of part 1” (see error type 1).

1. **Reporting contributory causes in Part 1**

Contributory causes are the conditions that were present at the time of death but were not included in the chain of events leading to death. Such conditions should be reported in Part 2 of the death certificate.(4)

1. **Reporting underlying cause in Part 2**

The potential underlying cause of death should be reported in the lowest used line of Part 1 of the death certificate.(4) Reporting the underlying cause of death in Part 2 may lead to erroneous underlying cause of death selection.

1. **Unspecified neoplasms**

Site, morphology and the behaviour of neoplasms are important information that should be reported on the death certificate. In the absence of such information, it is likely that the neoplasm will be coded to an ill-defined category such as C80 - malignant neoplasms of ill-defined sites , or D48 - neoplasms of uncertain or unknown behaviour of other and unspecified sites, neither of which are of significant public health value.

1. **Poorly defined external cause of death**

In certifying deaths due to injuries, poisoning and other external causes, the circumstances of the accident or violence (external cause) should be reported as the underlying cause of death. This external cause should be described in as much detail as possible. (1, 4) In the absence of such information, the underlying cause will be coded to unspecified categories such as V99, unspecified transport accident, or X59, exposure to unspecified factor, etc.(4)

**References**

1. Gamage USH, Chowdhury, H, Li, H, Koralage, M B, Mikkelsen, L, Hart, J, Lopez, A, McLaughlin, D, Hudson, S, Sarmiento, C, Bo, S, Kwa, V, Reeve, M. . Assessing the quality of death certificates: Guidance for the rapid tool.2020 02 01 2021. Available from: <https://crvsgateway.info/file/17055/62>.

2. Mikkelson L, Richards, N, Lopez, AD. . Redefining ‘garbage codes’ for public health policy: Report on the expert group meeting,2017. Available from: <https://crvsgateway.info/file/16948/276>.

3. Naghavi M, Richards N, Chowdhury H, Eynstone-Hinkins J, Franca E, Hegnauer M, et al. Improving the quality of cause of death data for public health policy: are all 'garbage' codes equally problematic? BMC Med. 2020;18(1):55.

4. WHO. International Statistical Classification of Diseases and Related Health Problems. 10th Revision. Vol. 2. . Geneva: World Health Organization; 1993.
