# Supplementary material for: The impact of errors in medical certification on the accuracy of the underlying cause of death
Source: PLoS One. 2021 Nov 8;16(11):e0259667. doi: 10.1371/journal.pone.0259667 (PMC8575485; doi:10.1371/journal.pone.0259667)
Supplement: S3 Text — (DOCX) [file pone.0259667.s003.docx]

**S3 Text. Business Rules used**

1. **Reporting multiple causes in a single line of Part 1**

Movement of causes based on the lowest line used:

1 d lowest used line

- Move 1 d to 1 c (50% as the first cause on 1c; 50% as the second cause on 1c)
- Move 1 c to 1 b (50% as the first cause on 1b; 50% as the second cause on 1b), adjust lower causes upwards
- Move 1 b to 1 a (50% as the first cause on 1a; 50% as the second cause on a), adjust lower causes upwards

Each move made in an equal number of certificates (randomly selected).

1 c Lowest used line

- Move 1 c to 1 b (50% as the first cause on 1b; 50% as the second cause on 1b)
- Move 1 b to 1 a (50% as the first cause on 1a; 50% as the second cause on 1a), adjust lower causes upwards

Each move made in an equal number of certificates (randomly selected).

1 b Lowest used line

- Move 1 b to 1a in all such certificates (50% as the first cause on 1a; 50% as the second cause on 1a)

1. **Incorrect or clinically improbable sequence**

Causes re-ordered within Part 1.

Certificates with 2-5 lines used

- Re-order causes randomly.

Certificates with only one line used in Part 1

- No change

1. **Ill-defined underlying cause**

The condition in the lowest used line replaced with:

1. Symptom or sign related to the condition reported in the lowest used line

2 Mode of dying related to the condition reported in the lowest used line

3. Intermediate cause – remove the condition in the lowest used line, leaving the intermediate cause as the underlying cause

1, 2 and 3 are randomly allocated using the proportions in the table below to deaths in high, medium and low socio-demographic index (SDI) countries. These proportions are calculated as the average from 20 countries used in the analysis by Iburg et al.(1)

**S3 Table. Proportions of ill-defined causes by type and SDI, 20 countries**

| **SDI countries** | **Symptoms/ signs (R00-R99)** | **Mode of dying** | **Intermediate causes** |
| --- | --- | --- | --- |
| High | 28.0% | 2.3% | 69.7% |
| Medium | 31.2% | 4.1% | 64.7% |
| Low | 20.0% | 4.9% | 75.1% |

1. **Competing causes in part 1**

Pre-identified set of competing causes introduced into Part 1. Competing causes are from the main body systems involved with most frequent causes of deaths e.g. Cardiovascular, Respiratory, Endocrine, Infectious and parasitic etc.

A cause from another body system will be introduced as follows:

- If three lines used – Competing cause inserted into Part 1 location (before 1a, between 1a and 1b, between 1b and 1c, after 1c) in equal proportions (selected randomly). Adjust lower causes accordingly.
- If two lines used – Competing cause inserted into Part 1 location (before 1a, between 1a and 1b, after 1b) in equal proportions (selected randomly). Adjust lower causes accordingly.
- If only 1a is used – Competing cause inserted into Part 1 location (before 1a, after 1a) in equal proportions (selected randomly). Adjust lower causes accordingly
- If four lines used, do not make any change.

1. **Reporting contributory causes in part 1**

The condition reported in Part 2 moved randomly into the lines of Part 1.

If three lines used – Part 2 cause inserted into Part 1 location (before 1a, between 1a and 1b, between 1b and 1c, after 1c) in equal proportions (selected randomly). Adjust lower causes accordingly.

If two lines used – Part 2 cause inserted into Part 1 location (before 1a, between 1a and 1b, after 1b) in equal proportions (selected randomly). Adjust lower causes accordingly.

If only 1 a is used – Part 2 cause inserted into Part 1 location (before 1a, after 1a) in equal proportions (selected randomly). Adjust lower causes accordingly.

Do not insert if four lines used.

1. **Reporting underlying cause in part 2**

The condition reported in the lowest used line of Part 1 moved to the first condition in Part 2.

Do not move where only 1 cause reported in Part 1.

1. **Unspecified neoplasm**

Underlying cause of death malignant neoplasm replaced by the code for the ‘Malignant neoplasms without specification of site’ C80.

Benign neoplasms replaced with D36.9 Benign neoplasms of unspecified site

1. **Missing information about external causes**

Information about external causes was removed from the records of deaths where external causes was the underlying cause.

1. **Illegibility**

Two responses by coders to illegible entries are assessed:

1. Skip illegible entry and assign no code
2. Assign the code R99 – other ill-defined or unknown causes of mortality

Other types of responses to illegible entries are not assessed:

1. Misread the entry and assign a wrong ICD code
2. Attempt to replace the entry with a plausible replacement based on the other causes reported

In each certificate, either R99 or ‘no code’ (50% each, randomly chosen) was entered into one line of the certificate. The line was randomly chosen according to the number of lines used on the certificate (e.g. if 3 lines in Part 1 used, the line is 33% likely to be 1a, 33% to be 1b and 33% to be 1c).

1. **Absent or erroneous time intervals**

Time intervals were not available in the data set used for simulation. We introduced incorrect time intervals into error free records as either inappropriate time intervals for individual causes and cases or incorrect time sequences.

**References**

1. Iburg KM, Mikkelsen L, Adair T, Lopez AD. Are cause of death data fit for purpose? evidence from 20 countries at different levels of socio-economic development. PLOS ONE. 2020;15(8):e0237539.
