# Supplementary material for: The impact of errors in medical certification on the accuracy of the underlying cause of death
Source: PLoS One. 2021 Nov 8;16(11):e0259667. doi: 10.1371/journal.pone.0259667 (PMC8575485; doi:10.1371/journal.pone.0259667)
Supplement: S1 Table — (DOCX) [file pone.0259667.s004.docx]

**S1 Table. Age, sex and cause distribution of MCCODs in final sample (%), low, middle, high SDI countries and all**

| **Low (N=972)** | | | | | | |
| --- | --- | --- | --- | --- | --- | --- |
| *Male* | Group I | CVD | Cancers | Other NCD | Injuries | Total |
| 0-4 | 12.7 | 0.0 | 0.1 | 1.4 | 0.6 | 14.8 |
| 5-44 | 3.9 | 0.9 | 0.4 | 1.5 | 3.1 | 9.9 |
| 45-64 | 3.0 | 3.3 | 1.3 | 3.0 | 1.1 | 11.7 |
| 65-84 | 3.0 | 4.3 | 1.5 | 4.9 | 0.8 | 14.6 |
| 85+ | 0.6 | 0.8 | 0.1 | 1.0 | 0.1 | 2.7 |
| Total | 23.1 | 9.4 | 3.5 | 11.9 | 5.8 | 53.7 |
| *Female* | Group I | CVD | Cancers | Other NCD | Injuries | Total |
| 0-4 | 10.8 | 0.0 | 0.0 | 1.4 | 0.5 | 12.8 |
| 5-44 | 4.1 | 0.7 | 0.6 | 0.9 | 1.2 | 7.6 |
| 45-64 | 2.2 | 2.4 | 1.6 | 2.2 | 0.5 | 8.8 |
| 65-84 | 3.2 | 4.5 | 1.3 | 4.5 | 0.6 | 14.2 |
| 85+ | 0.7 | 0.9 | 0.1 | 1.0 | 0.1 | 2.9 |
| Total | 21.0 | 8.5 | 3.7 | 10.1 | 3.0 | 46.3 |
| **Middle (N=971)** | | | | | | |
| *Male* | Group I | CVD | Cancers | Other NCD | Injuries | Total |
| 0-4 | 1.6 | 0.0 | 0.0 | 0.4 | 0.2 | 2.3 |
| 5-44 | 1.2 | 1.0 | 0.6 | 1.2 | 3.4 | 7.5 |
| 45-64 | 1.3 | 5.5 | 3.7 | 3.5 | 1.8 | 15.8 |
| 65-84 | 1.6 | 10.5 | 4.9 | 6.7 | 1.0 | 24.8 |
| 85+ | 0.5 | 2.9 | 0.5 | 2.2 | 0.2 | 6.3 |
| Total | 6.4 | 19.9 | 9.8 | 14.0 | 6.6 | 56.6 |
| *Female* | Group I | CVD | Cancers | Other NCD | Injuries | Total |
| 0-4 | 1.3 | 0.0 | 0.0 | 0.3 | 0.1 | 1.8 |
| 5-44 | 0.9 | 0.5 | 0.6 | 0.6 | 0.9 | 3.6 |
| 45-64 | 0.8 | 2.9 | 2.6 | 2.1 | 0.6 | 9.0 |
| 65-84 | 1.5 | 8.9 | 3.2 | 5.9 | 0.8 | 20.3 |
| 85+ | 0.7 | 4.1 | 0.6 | 3.0 | 0.3 | 8.8 |
| Total | 5.4 | 16.4 | 7.0 | 11.8 | 2.8 | 43.4 |
| **High (N=952)** | | | | | | |
| *Male* | Group I | CVD | Cancers | Other NCD | Injuries | Total |
| 0-4 | 0.2 | 0.0 | 0.0 | 0.1 | 0.0 | 0.3 |
| 5-44 | 0.1 | 0.3 | 0.2 | 0.7 | 1.2 | 2.5 |
| 45-64 | 0.3 | 2.5 | 3.5 | 2.2 | 1.1 | 9.6 |
| 65-84 | 1.2 | 7.6 | 8.9 | 6.3 | 1.1 | 25.0 |
| 85+ | 0.9 | 5.4 | 2.4 | 4.4 | 0.5 | 13.7 |
| Total | 2.7 | 15.8 | 15.0 | 13.8 | 3.8 | 51.1 |
| *Female* | Group I | CVD | Cancers | Other NCD | Injuries | Total |
| 0-4 | 0.1 | 0.0 | 0.0 | 0.1 | 0.0 | 0.2 |
| 5-44 | 0.1 | 0.1 | 0.3 | 0.3 | 0.3 | 1.2 |
| 45-64 | 0.2 | 1.1 | 2.6 | 1.3 | 0.3 | 5.5 |
| 65-84 | 0.8 | 6.0 | 5.9 | 5.8 | 0.6 | 19.1 |
| 85+ | 1.5 | 9.8 | 2.7 | 8.3 | 0.7 | 23.0 |
| Total | 2.7 | 16.9 | 11.6 | 15.8 | 2.0 | 48.9 |
| **All (N=1592)** | | | | | | |
| *Male* | Group I | CVD | Cancers | Other NCD | Injuries | Total |
| 0-4 | 7.7 | 0.0 | 0.1 | 0.9 | 0.4 | 9.0 |
| 5-44 | 2.4 | 0.6 | 0.4 | 0.9 | 2.1 | 6.4 |
| 45-64 | 1.8 | 3.3 | 2.3 | 2.1 | 1.1 | 10.6 |
| 65-84 | 1.8 | 6.4 | 5.3 | 4.1 | 0.6 | 18.3 |
| 85+ | 0.6 | 3.2 | 1.4 | 2.6 | 0.3 | 8.2 |
| Total | 14.3 | 13.6 | 9.5 | 10.7 | 4.5 | 52.5 |
| *Female* | Group I | CVD | Cancers | Other NCD | Injuries | Total |
| 0-4 | 6.6 | 0.0 | 0.0 | 0.9 | 0.3 | 7.8 |
| 5-44 | 2.5 | 0.4 | 0.4 | 0.6 | 0.8 | 4.6 |
| 45-64 | 1.3 | 1.8 | 1.6 | 1.3 | 0.4 | 6.3 |
| 65-84 | 1.9 | 5.4 | 3.5 | 3.6 | 0.5 | 14.9 |
| 85+ | 0.9 | 5.8 | 1.6 | 5.0 | 0.4 | 13.8 |
| Total | 13.3 | 13.4 | 7.1 | 11.3 | 2.4 | 47.5 |
