# Supplementary material for: The impact of errors in medical certification on the accuracy of the underlying cause of death
Source: PLoS One. 2021 Nov 8;16(11):e0259667. doi: 10.1371/journal.pone.0259667 (PMC8575485; doi:10.1371/journal.pone.0259667)
Supplement: S2 Table — (DOCX) [file pone.0259667.s005.docx]

**S2 Table. Chance-Corrected Concordance for ill-defined UCOD error, by number of causes reported on death certificate and SDI level**

|  |  | **SDI level** | | |
| --- | --- | --- | --- | --- |
| **Number of causes reported** | **All** | **High** | **Middle** | **Low** |
| Ill-defined UCOD: 1 cause | 0.353 | 0.138 | 0.200 | 0.457 |
| Ill-defined UCOD: >1 cause | 0.454 | 0.385 | 0.401 | 0.532 |
