# Supplementary material for: The impact of errors in medical certification on the accuracy of the underlying cause of death
Source: PLoS One. 2021 Nov 8;16(11):e0259667. doi: 10.1371/journal.pone.0259667 (PMC8575485; doi:10.1371/journal.pone.0259667)
Supplement: S3 Table — (DOCX) [file pone.0259667.s006.docx]

**S3 Table. CSMF Accuracy and Weights by error type and SDI level**

| **Error type** | **All** | | **SDI level** | | | | | |
| --- | --- | --- | --- | --- | --- | --- | --- | --- |
|  |  |  | **High** | | **Middle** | | **Low** | |
|  | **CSMF Accuracy** | **Weight** | **CSMF Accuracy** | **Weight** | **CSMF Accuracy** | **Weight** | **CSMF Accuracy** | **Weight** |
| Contributory cause in Part 1 | 0.928 | 0.072 | 0.928 | 0.072 | 0.917 | 0.083 | 0.934 | 0.066 |
| Underlying cause in Part 2 | 0.907 | 0.093 | 0.892 | 0.108 | 0.896 | 0.104 | 0.902 | 0.098 |
| Multiple causes per line | 0.959 | 0.041 | 0.951 | 0.049 | 0.953 | 0.047 | 0.959 | 0.041 |
| Incorrect sequence | 0.961 | 0.039 | 0.949 | 0.051 | 0.955 | 0.045 | 0.962 | 0.038 |
| Ill-defined UCOD | 0.675 | 0.325 | 0.613 | 0.387 | 0.641 | 0.359 | 0.734 | 0.266 |
| Competing cause in Part 1 | 0.857 | 0.143 | 0.902 | 0.098 | 0.882 | 0.118 | 0.819 | 0.181 |
| Illegibility (R99 or blank line) | 0.811 | 0.189 | 0.778 | 0.222 | 0.794 | 0.206 | 0.834 | 0.166 |
| Time interval | 0.886 | 0.114 | 0.872 | 0.128 | 0.878 | 0.122 | 0.888 | 0.112 |
| External cause* | 0.944 | 0.056 | 0.953 | 0.047 | 0.924 | 0.076 | 0.928 | 0.072 |
| Neoplasms* | 0.856 | 0.144 | 0.775 | 0.225 | 0.859 | 0.141 | 0.927 | 0.073 |

Very High Impact (Red): Weight>0.25. High Impact (Brown): Weight 0.10<0.25. Medium Impact (Yellow): Weight 0.05<0.10. Low Impact (Green): Weight<0.05.

* External cause and neoplasms are error types only related to specific types of causes of death, and their importance will depend on the percentage of deaths that are due to each of these causes. Hence, these are not included in the error impact categories.
