# Supplementary material for: The impact of errors in medical certification on the accuracy of the underlying cause of death
Source: PLoS One. 2021 Nov 8;16(11):e0259667. doi: 10.1371/journal.pone.0259667 (PMC8575485; doi:10.1371/journal.pone.0259667)
Supplement: Attachment — Submitted filename: Response to reviewers.docx [file pone.0259667.s009.docx]

**Responses to reviewers’ comments**

 Review Comments to the Author

We thank the Editor for providing us the opportunity to respond to the reviewers’ valuable comments. Please find our responses below in red. Line numbers refer to the clean version of the manuscript.

Reviewer #1: This is an extremely important topic.
We thank the reviewer for their comments. Please find our responses below in red. Line numbers refer to the tracked version of the manuscript.

We would like to clarify that our study assesses the impact on the accuracy of cause of death statistics of errors made by physicians in completing the Medical Certificate of Cause of Death, as according to the WHO standard guidelines in certification. We did not specifically assess the diagnostic accuracy of the diseases and conditions reported on the certificate against an autopsy. Hence, the “error-free” death certificates are those where the physician has made no errors in completing the death certificate, based on the WHO standard guidelines in certification. It is likely that many of these “error-free” death certificates would not have the “true” cause of death, as assessed when compared with an autopsy, for various reasons (e.g. diagnostic limitations, biases of the physician, etc). Our assessment of the accuracy of cause of death statistics compares, for each death in the database, the underlying cause of death (after the application of ICD coding rules) where there is each type of certification error versus the underlying cause of death where there is no error on the death certificate.

To reflect this, we have amended the title of the manuscript (removing the term “diagnostic”) and also clarified the specific nature of the study and methods used in these places:

- Abstract Lines 26-27 and Line 33-34
- Introduction Line 69
- Methods Lines 111-115, 171-173, 185-87
- Discussion Lines 359-61

Rejection causes:
Major:
1. Multiple recognized (recent and high impact) Major and Minor diagnosis errors systems have been published but ignored.

We based our categorisation of certification error types on those identified as the most common according to published studies. The reviewer has stated that there are other diagnosis errors systems, however the errors that we are focusing on are errors in *completing* the death certificate according to WHO guidelines and how these impact on the accuracy of causes of death (after the application of ICD coding rules), rather than errors specifically related to *diagnostic accuracy* of the diseases and conditions reported on the certificate. We note that a recent article by Schuppener et al. (Death certification: errors and interventions. Clinical Medicine & Research, 2020;18(1):21-6) identified “Wrong COD” as an error type (we have added a description of this study in Lines 92-99). However, this kind of error identification and categorization is only possible when the death certificate entries are validated with medical records or autopsy findings, and is not applicable to assessment of errors in completing the MCCOD.

2. The basis of the study is the impact of the errors. This can NOT be calculated if the true cause is UNKNOWN. If there is a focus on reading recent (and old) publications the authors will understand that this is the reason data and statistics from current death certificates can NOT be used. Regardless what sophisticated statistics are used with "garbage" data, it will still result in erroneous results, discussion and conclusion (garbage in, garbage out).

As mentioned above, our aim was to measure the impact of each different type of error in completing the death certificate (according to WHO guidelines) on the resultant cause of death (after application of ICD coding rules). We do this by comparing the cause of death of the death certificates with an error against those without an error, using the metric of chance-corrected concordance. However, we do recognise that we do not know the true cause of each death, which may be impacted by diagnostic equipment, physician biases or training, information available for the physician, etc. We don’t necessarily believe the error-free certificates have the ‘true’ cause of death (as compared with an autopsy) but the cause of death where none of certification errors are present. We have clarified this issue in Lines 111-115 and 359-61.

We do not agree however that data and statistics from current death certificates cannot be used. In fact, they are used widely, including by the WHO, Global Burden of Disease Study and numerous national statistics offices. There are established methods to assess the extent and severity of garbage codes and reallocate these to non-garbage causes (see Naghavi M et al. Improving the quality of cause of death data for public health policy: are all 'garbage' codes equally problematic? BMC Med. 2020;18(1):55, and Naghavi M, et al. Algorithms for enhancing public health utility of national causes-of-death data. Popul Health Metr. 2010;8:9). We also recognise that it is not possible to conduct an autopsy on all deaths, so physician-completed death certificates are the primary source of information on causes of death. We also recognise that there should be continued efforts to improve training of physicians in death certification is important to ensure that the data are as useful for policy as possible.

Minor:
1, Grammar and typo errors (multiple). Many that would be highlighted and corrected by word or google documents

We have amended grammar and typo errors in multiple places.

2. Old references (except those by authors. Self referencing (while excluding others) gives a false impression of importance without recognizing others

We didn’t intend to create a false impression of importance. We have now added more recent references of Schuppener et al, 2020 (reference #13, described in lines 92-97) and Brooks & Reed, 2015 (reference #12, Lines 73, 84). We note that other references we have used are relatively recent: McGiven 2017, Flippatos 2016, Miki 2018, Crandll 2020, Azim 2014.

3. Repetition of reference 14 and 15 (30 and 32)

Thank you for identifying these issues - we have deleted these duplicate references.

Reviewer #2: This is a very interesting paper dealing with an essential aspect of public health monitoring: the impact of errors in medical certification on the diagnostic accuracy of the underlyng cause of death. This is the first study I read about measuring the relative impact of various common medical certification errors on the UCOD and that gives important results to improve medical certification.

We thank the reviewer for providing valuable comments on the manuscript. Please find our responses below in red. Line numbers refer to the tracked version of the manuscript.

Following are my comments:
1. The error-free MCCODs were obtained from the United States’ (US) Multiple Cause of Death data file for 2017 and after that a sample of 1592 MCCODs were used. It would be important to know how many MCCODs were available in the US data file.

There were a total of 2,820,034 MCCODs in the UCOD data file in 2017 – we have now included this figure in Line 121. We selected a sample of MCCODs because each MCCOD needed to be individually screened for errors by trained MCCOD certifiers so that they could select the error-free certificates.

2. About the reporting of an ill-defined condition as the UCOD error, I would like to know if there was more impact in the MCCODs that had only one line written in part 1 or if there were other ill-defined cause of death in all the other lines of part 1.

The reviewer raises a good point. We conducted a further analysis of whether there was greater impact on MCCODs of the ill-defined error with only one cause written in Part 1 of the death certificate compared with more than one cause. The results shown in the table below show that the chance-corrected concordance for the ill-defined error with only one cause reported is 0.353 compared with 0.454 for more than one error. Notably, the chance-corrected concordance for the ill-defined error with only one cause reported is particularly low in high SDI (0.138) and middle SDI (0.200) countries, but with less of an impact in low SDI countries where the problem of only one cause being reported may be more pronounced.

We have included this table in S2 Table and briefly described the results in Lines 229-231 and 286-287.

**S2 Table. Chance-Corrected Concordance for ill-defined UCOD error, by number of causes reported on death certificate and SDI level**

|  |  | **SDI level** | | |
| --- | --- | --- | --- | --- |
| **Number of causes reported** | **All** | **High** | **Middle** | **Low** |
| Ill-defined UCOD: 1 cause | 0.353 | 0.138 | 0.200 | 0.457 |
| Ill-defined UCOD: >1 cause | 0.454 | 0.385 | 0.401 | 0.532 |

3. About the illegibility error, you mentioned that if there were an illegible line, the coder would skip the illegible entry and not assign a code, how do you difference that from a blank space left by the medical certifier.

In our simulation of the illegibility error, one assumption we made was that the coder would skip the illegible entry and not assign a code. In practice, this may be the same as if the certifier did leave a blank space. Leaving a blank space will generally cause a lesser impact on coding and the selection of the underlying cause than the removal of a condition from the certificate due to illegibility. Blank spaces are usually ignored during the coding process, but the inability to code due to illegibility means a removal of a condition which had been originally reported by the certifier. The latter causes a higher impact on the selection of underlying cause than blank spaces.

4. It would have been important to analyse the use of abbreviations, at least for the countries using Iris coding.

We agree that it would have been valuable to analyse the use of abbreviations, however we were unable to do so because we were using a digitised database and we could not identify a plausible means of simulating this error. We have mentioned this issue already in Lines 353-355. However, any abbreviation can be included into the Iris dictionary using standardization tables, which can lessen the impact of abbreviations on cause of death statistics.

Reviewer #3: 1.A well-written, neatly structured manuscript
2.The manuscript is technically sound and the data does support the conclusions
3. Statistical analysis and explanation of results is clear
4. Conclusion needs to be redone to incorporate lot more information

We thank the reviewer for their valuable comments.

1. Since the study uses error-free MCCODS from US database, generalizing the results across the globe (primarily in medium and low income countries) would be wrong. In fact, the study states that the impact of errors on selection of UCOD is lesser than expected. if the sample was MCCODs collected from diverse countries the results would be different altogether. The authors need to emphasize on this point lot more both in the discussion and conclusion

We would like to clarify how we sought to generalise the results from our study to middle and low income (Socio-Demographic Index or SDI) countries. In the study, we selected samples of MCCODs that represent the age, sex and cause profile of low, middle and high SDI countries, to provide results that estimate how each error type would impact cause of death accuracy across the different epidemiological profiles present in the world. For example, the Low SDI sample has a higher proportion of deaths from infectious diseases compared with the High SDI sample (see S1 Table). Unfortunately, we were unable to use a similar database from a low- or middle-income country. In the discussion, we have already stated in Lines 325-327 that “Additionally, the relatively high quality of the error-free certificates, obtained from a US database which is of higher quality than databases in many low- and middle-income countries, may also have played a part in the lower than expected impact of errors.” We have however now added in the Discussion in Lines 332-334 that the magnitude of the results may be different if we were able to use data from a low- or middle-income country. We have also mentioned this overall limitation in the Conclusion (Lines 381-383).

3. Though the list of errors in death certification are similar across countries, their impact on diagnostic accuracy of UCOD is varied especially in medium and low income countries. Emphasis on this differentiation is not evident in the manuscript.

We primarily based our interpretation of our results in terms of their differential impact on high, middle and low SDI countries from Table 1. This shows that there are considerable differences in the chance-corrected concordance of the ill-defined error, especially where only one cause is reported in the MCCOD (see new S2 Table and Lines 229-231). Other clear differences are found for the unspecified neoplasms error, which is already reported in Lines 234-235. However, for none of the other errors is there a clear difference in the Chance-corrected concordance or Weight (and impact category) between any of the SDI levels, so we believe that it is not necessary to report these.

4. As mentioned in the limitation of the study, the impact of combined errors is not studied. The author needs to articulate much further the advantages of studying individual errors

The reviewer makes a good point. As we have mentioned in Lines 338-349, the main advantages of analysing individual errors is that it allows individual measurement of the impact of each error without being biased by the presence of other errors, that we do not need to make assumptions of the composition or combination of errors on MCCODs and that it does not require using a vast number of possible combination of causes. We have now expanded on the last of these issues in Lines 340-345, specifically that:

“Assessing the diagnostic impact of clusters of errors would however require a much larger data set and would involve vast possible combinations of two or more of the nine errors. For example, assessment of the ill-defined error may need to be conducted with one of each of the other errors, different combinations of each of the other errors, up to all eight other errors; this would make disentangling the relative importance of each error a major methodological challenge that is beyond the scope of this study.”

5. Conclusion needs to be redone to incorporate lot more information

We are unsure which specific information should be added to the Conclusion, keeping in mind that it should only be relatively brief. However, in the Conclusion we have now included information on the overall limitation of the use of the selection of samples of MCCODs from the US database that represent the age, sex and cause profile of low, middle and high SDI countries due to no alternate database being available from low- or middle-income countries.

**Do you want your identity to be public for this peer review?** For information about this choice, including consent withdrawal, please see our [Privacy Policy](https://www.plos.org/privacy-policy).

Reviewer #1: No

Reviewer #2: **Yes:** Janet Miki

Reviewer #3: **Yes:** Dr Kavya Rangaswamy
