# Supplementary material for: The impact of errors in medical certification on the accuracy of the underlying cause of death
Source: PLoS One. 2021 Nov 8;16(11):e0259667. doi: 10.1371/journal.pone.0259667 (PMC8575485; doi:10.1371/journal.pone.0259667)
Supplement: Attachment — Submitted filename: Response to reviewers final.docx [file pone.0259667.s010.docx]

**PONE-D-21-14961R1: The impact of errors in medical certification on the accuracy of the underlying cause of death**

**Responses to reviewers’ comments**

We thank the Editor for the opportunity to respond to the reviewers’ comments. Please find below our responses. All line numbers refer to the document with tracked changes.

Reviewer #5: A well written and novel approach to estimating the extent to which MCCODS errors affect the UCOD. Kindly find herein my comments on the article:

We thank the reviewer for their valuable comments. Please find below our responses. All line numbers refer to the document with tracked changes.

• Line 43-42 : The authors can mention what errors, if any, came under the “Low impact error type” as the paragraph mentions the classification of error types into very high, high, medium, and low.

We have now added that the low impact errors were multiple causes per line and incorrect sequence.

• Line 49 : Explanation of what Iris stands for, (the automated coding software), would give an uninitiated reader a better understanding as it may be a new term for many young physicians.

Iris is an automated mortality coding software – we now state this in Line 35.

• Line 60 : The S1 text reference only shows the guidelines for filling out the WHO MCCOD. However, there should also be a reference for the guidelines for Coding mentioned here as it has been specifically mentioned in lines 59-60.

We have now added Reference 1 (WHO ICD – 10 vol 2) in Line 62.

• Line 72 : Does the “very clear rules” mentioned here refer to lines 70-71? If not, then does it refer to S1 text reference? Or is it mentioned among the other references (4-13)? More clarity is needed on this.

These “very clear rules” are those in WHO ICD – 10 vol 2 (reference #1) – we have now stated this in Lines 74-75.

• S2 text : “Reporting multiple causes in a single line of part 1” and “Reporting competing causes in part 1” appear at first glance to be separate entities. So, does that imply that the multiple causes in the single line are not mutually exclusive but equally possible? And the competing causes of part 1 may also be in a single line. Which type of error would it then come under? As the result is essentially wrongly coding 1 of the causes listed as the UCOD. A better explanation/differentiation between the two types of errors would be helpful.

Competing causes are mutually exclusive conditions reported in different lines of part 1. Multiple causes in a single line are considered a different error category, irrespective of whether the multiple causes in the single line are mutually exclusive or not. If two competing causes are reported on a single line in Part 1, then we classified the error as “Reporting multiple causes in a single line of part 1”. We have clarified this in S2 Text, certification error type 7 “Reporting competing causes in Part 1”.

• Line 123 and footnote 1 : Though the explanation for how SDI is calculated is mentioned, since the authors have mentioned that it is used by Global burden of disease study, a reference to a GBD capstone article/ related GBD reference may be included.

We have added a reference (27) in Line 129.

• Line 136 : Would the decision to include the MCCODs in multiple SDI groups not affect the results? The rationale for including them in multiple SDI groups needs to be mentioned for better clarity. The authors also need to clarify here whether MCCODS with “unspecified neoplasms” error and “poorly defined external causes” error were also out in multiple SDI groups or not.

The decision to include MCCODs in multiple SDI groups is not expected to affect the results because deaths with the same causal sequence would occur in high, medium and low SDI countries. We included the same MCCODs in multiple SDI groups because the process for identifying error-free death certificates was conducted manually and was very time-intensive; hence, this reduced the amount of time needed to identify error-free death certificates. We have now clarified this in Lines 139-141. The “unspecified neoplasms” error and “poorly defined external causes” error were both simulated on MCCODs that were included in multiple SDI groups.

• Fig 1 : SDI categories are not labelled.

The SDI categories in Figure 1 are now labeled.

• Lines 272-273 : Though one of the stated goals was “to monitor trends in the quality of medical certification”, subsequent sections fail to elaborate on this. Though there is the mention of the use of Iris in mainly high SDI countries and the possibility of manual coding causing more errors, there isn’t enough discussion to correlate the study findings and the “quality of medical certification” in other countries.

This sentence should have read “…to enable the monitoring of trends in the quality of medical certification”. We have subsequently modified the existing sentence in Lines 318-320 to be “The weights also allow development of a composite index of certification quality which can be used to monitor trends and differentials in overall certification practices from a database of MCCODs.”

• Lines 279-281 : More details need to be mentioned regarding situations where epidemiological and other public health factors are considered in the UCOD selection. Since these coding rules are mentioned as the main reason for mitigating errors, the situations where they are applied instead of using the condition initiating the causal sequence needs to be elaborated. And if according to the coding rules, some other condition is selected as the UCOD and not the condition initiating the causal sequence, should it still be considered as an error for the purpose of this study?

In mortality coding, whether a causal relationship is considered acceptable or not for mortality coding is established not only on a medical assessment but also on epidemiological and public health considerations. Therefore, a medically acceptable relationship might be listed as unacceptable in the coding instructions because a later step in the sequence is more important from a public health point of view. However, a medically acceptable sequence was never considered as an error for the purposes of this study.

• Line 296 : A brief explanation of Coding rules SP4 and SP5 is needed even though a reference for the same is given (24). This is because of the importance of these rules in understanding why the effect of published “major errors” may not be as high as expected.

Usually, the first cause reported in the lowest used line of part 1 is the underlying cause of death meant by the physician. In an incorrectly reported sequence, the first condition reported in the lowest used line may not explain all the conditions reported above. Rule SP4 allows the coder to search for another possible sequence ending in the first mentioned condition (terminal condition) in part 1 and select the originating cause of that sequence as the tentative starting point. Further, when there is no sequence ending with the terminal condition, the rule SP5 allows the coder to select the terminal condition also as the tentative starting point. We have added this information in a footnote (footnote 2).

Reviewer #6: Dear Authors,

The authors have satisfactorily addressed all the queries by the previous reviewers. This article is very relevant in the present scenario of COVID pandemic. The authors are requested to address the below queries .

We thank the reviewer for their valuable comments. Please find below our responses. All line numbers refer to the document with tracked changes.

Abstract:

• Line 32 and 33: The author needs to clarify whether “ 1592- error free reports” selected were “ either the death occurred in the medical institution or non- institutional death”, since WHO has recommended two types of Forms specifically ie FORM 4 and FORM 4A.

The 1592 error-free MCCODs were US registered deaths that occurred in all settings - i.e. in hospital, at home, in public places, etc. We have now clarified this Lines 124-125.

Discussion:

• Line 328: The author needs to write the type of version and make of “ Iris automated coding software” and the calibration to avoid errors in coding.

Iris version 5.7 was used for coding – this is now mentioned in Line 164.
