# Supplementary material for: The impact of errors in medical certification on the accuracy of the underlying cause of death
Source: PLoS One. 2021 Nov 8;16(11):e0259667. doi: 10.1371/journal.pone.0259667 (PMC8575485; doi:10.1371/journal.pone.0259667)
Supplement: Attachment — Submitted filename: Dr Kavya R reviewer comments.docx [file pone.0259667.s008.docx]

1. Since the study uses error-free MCCODS from US database, generalizing the results across the globe (primarily in medium and low income countries) would be wrong. In fact, the study states that the impact of errors on selection of UCOD is lesser than expected. if the sample was MCCODs collected from diverse countries the results would be different altogether. The authors need to emphasize on this point lot more both in the discussion and conclusion

3. Though the list of errors in death certification are similar across countries, their impact on diagnostic accuracy of UCOD is varied especially in medium and low income countries. Emphasis on this differentiation is not evident in the manuscript

4. As mentioned in the limitation of the study, the impact of combined errors is not studied. The author needs to articulate much further the advantages of studying individual errors

5. Conclusion needs to be redone to incorporate lot more information
